# Supplementary material for: Socio-economic, demographic, and behavioural determinants of women’s empowerment in Mozambique
Source: PLoS One. 2021 May 28;16(5):e0252294. doi: 10.1371/journal.pone.0252294 (PMC8162630; doi:10.1371/journal.pone.0252294)
Supplement: S1 Table — (DOCX) [file pone.0252294.s001.docx]

**S1 Table. Indicators of empowerment identified in Mozambique DHS 2015.**

| **Selected indicators** | **Codes** | **Recoding** |
| --- | --- | --- |
| Who usually decides on women’s health care? | Respondent alone=1; Respondent and husband/partner=2; husband/partner alone=4; Someone else=5; Other=6 | Respondent alone =1; Jointly= 0; Partner or other alone­= -1 |
| Who usually decides on large purchases for the household? | Respondent alone=1; Respondent and husband/partner=2; husband/partner alone=4; Someone else=5; Other=6 | Respondent alone =1; Jointly= 0; Partner or other alone­= -1 |
| Who usually decides on visits to family and friends? | Respondent alone=1; Respondent and husband/partner=2; husband/partner alone=4; Someone else=5; Other=6 | Respondent alone =1; Jointly= 0; Partner or other alone­= -1 |
| Is beating justified if wife goes out without telling husband? | No=0; Yes=1; Don’t know=8 | Not Justified=1; Don’t know=0; Justified =- 1 |
| Is beating justified if wife neglects the children? | No=0; Yes=1; Don’t know=8 | Not Justified=1; Don’t know=0; Justified =- 1 |
| Is beating justified if wife argues with husband? | No=0; Yes=1; Don’t know=8 | Not Justified=1; Don’t know=0; Justified =- 1 |
| Is beating justified if wife refuses to have sex with husband? | No=0; Yes=1; Don’t know=8 | Not Justified=1; Don’t know=0; Justified =- 1 |
| Is beating justified if wife burns the food? | No=0; Yes=1; Don’t know=8 | Not Justified=1; Don’t know=0; Justified =- 1 |
| Can a wife ask a husband/partner to use condom if he has STI? | No=0; Yes=1; Don’t know=8 | Yes= 1; Don’t know=0; No= -1 |
| Can a wife refuse sex? | No=0; Yes=1; Don’t know=8 | Yes= 1; Don’t know=0; No= -1 |
| Can a wife ask the husband/partner to use condom? | No=0; Yes=1; Don’t know=8 | Yes= 1; Don’t know=0; No= -1 |
