# Supplementary material for: Socio-economic, demographic, and behavioural determinants of women’s empowerment in Mozambique
Source: PLoS One. 2021 May 28;16(5):e0252294. doi: 10.1371/journal.pone.0252294 (PMC8162630; doi:10.1371/journal.pone.0252294)
Supplement: S2 Table — (DOCX) [file pone.0252294.s002.docx]

**S2 Table. Retained components’ factor loadings after orthogonal varimax rotation**

|  |  | **Components** | | |
| --- | --- | --- | --- | --- |
|  | **Indicator** | **Beliefs about violence against women** | **Decision-making** | **Control over sexuality and safe sex** |
| 1 | Who usually decides on the respondent’s health care | -0.0176 | 0.5967 | -0.0197 |
| 2 | Who usually decides on large purchases for the household | -0.0075 | 0.5611 | 0.0321 |
| 3 | Who usually decides on visits to family and friends | 0.0257 | 0.5714 | -0.0127 |
| 4 | Beating justified if wife goes out without telling husband | 0.4336 | -0.0167 | -0.0055 |
| 5 | Beating justified if wife neglects the children | 0.4591 | -0.0193 | -0.0210 |
| 6 | Beating justified if wife argues with husband | 0.4767 | -0.0145 | -0.0147 |
| 7 | Beating justified if wife refuses to have sex with husband | 0.4545 | -0.0103 | 0.0276 |
| 8 | Beating justified if wife burns the food | 0.4050 | 0.0266 | 0.0246 |
| 9 | Wife justified asking husband to use condom if he has STI | -0.0290 | 0.0259 | 0.5264 |
| 10 | Wife can refuse sex | -0.0154 | -0.0148 | 0.5981 |
| 11 | Wife can ask the husband to use condom | 0.0353 | -0.0068 | 0.6013 |
