# Supplementary material for: Socio-economic, demographic, and behavioural determinants of women’s empowerment in Mozambique
Source: PLoS One. 2021 May 28;16(5):e0252294. doi: 10.1371/journal.pone.0252294 (PMC8162630; doi:10.1371/journal.pone.0252294)
Supplement: Attachment — Submitted filename: PlosOnereview.docx [file pone.0252294.s003.docx]

| Comment | Page and Line # |
| --- | --- |
| Suggest defining empowerment and what is gap in measurement in Mozambique. Your aims listed lines 119-123 are very clear, something similar to this in the abstract would be useful. | Page 2, 37-42 |
| Please list your results in the abstract by listing each domain and related contextual factors systematically to clarify. | Page 2, 53-59 |
| This is one definition, should make it clear that it is the one you are using, but not the only conceptualization of empowerment. Recommend you see Gram et al., 2018  https://doi.org/10.1007/s11205-018-2012-2  Organising concepts of ‘women’s empowerment for measurement : a typology | Page 4, line 91 |
| Would be helpful to include a bit more about participants and data collection for the survey: ie; is this urban and rural areas? Who collected the data, how are they trained, and how was data collected (ie paper, electronic etc). What is inclusion/exclusion criteria for women to be included in the survey. Not necessary to include all, but some would provide more depth to those not familiar with DHS data. | Page 4 138-144 |
| Please cite the sentence of your empowerment definition. | Page 4 lines 150-152 |
| Please describe briefly rationale for combining regions in the way that was done. | Page 4 lines 173-174 |
| Methods would be strengthened with rationale for use of orthogonal rotation- ie were other rotations attempted? | Lines 200-206 |
| Table 1. Title should stand alone: can you add something else to make it clear what is being displayed? |  |
| Table 3 is very comprehensive---is there a way to simplify these results or break them up into other sections? Consider removing Table 1 or adding it as a supplement to make more space to share the results in Table 3 broken into separate tables. |  |
| Consider adding a sentence or two of literature that supports this finding. | Line 290 |
| Consider splitting this into two sentences: 1 indicating other research that indicates the individual characteristics that impact empowerment, and 2 how this played out in this sample in Mozambique. | Line 293-295 |
| Please provide citation for this. | Line 307-309 |
| Please look into work by Dr. Sidney Schuler indicating what happens longitudinally when gender norms are transgressed and consider including this. | Line 310-312 |
